# The political polarization of COVID-19 treatments among physicians and laypeople in the United States

**DOI:** 10.1073/pnas.2216179120

**Published:** 2023-02-08

**Authors:** Joel M. Levin, Leigh A. Bukowski, Julia A. Minson, Jeremy M. Kahn

**Affiliations:** ^a^Katz Graduate School of Business, University of Pittsburgh, Pittsburgh, PA 15260; ^b^Department of Critical Care Medicine, University of Pittsburgh, Pittsburgh, PA 15213; ^c^Kennedy School of Government, Harvard University, Cambridge, MA 02138; ^d^Department of Health Policy and Management, University of Pittsburgh, Pittsburgh, PA 15261

**Keywords:** belief polarization, medical decision-making, COVID-19, expert judgment

## Abstract

In the United States, liberals and conservatives disagree about facts. To what extent does expertise attenuate these disagreements? To study this question, we compare the polarization of beliefs about COVID-19 treatments among laypeople and critical care physicians. We find that political ideology predicts both groups’ beliefs about a range of COVID-19 treatments. These associations persist after controlling for a rich set of covariates, including local politics. We study two potential explanations: a) that partisans are exposed to different information and b) that they interpret the same information in different ways, finding evidence for both. Polarization is driven by preferences for partisan cable news but not by exposure to scientific research. Using a set of embedded experiments, we demonstrate that partisans perceive scientific evidence differently when it pertains to a politicized treatment (ivermectin), relative to when the treatment is not identified. These results highlight the extent to which political ideology is increasingly relevant for understanding beliefs, even among expert decision makers such as physicians.

A growing literature on political polarization has documented unexpected links between political ideology and beliefs that are unrelated to the principles of liberalism or conservatism (1, 2). During the COVID-19 pandemic, liberal and conservative Americans have disagreed sharply on matters such as the origins of the virus (3), the severity of the pandemic (4, 5), and the effectiveness of a range of interventions, including masking, distancing, vaccination, and drugs like hydroxychloroquine and ivermectin (3, 5–7). Such disagreements inhibit cooperation, fuel partisan antipathy, and threaten public health.

Prior work offers clues about the roots of this polarization: Partisans consume different information (4, 8, 9), evaluate the same information in different ways (10, 11), and often lack the tools (12) or motivation (13, 14) to discriminate between accurate and inaccurate claims. On these bases, we might expect beliefs about COVID-19 treatments to be dramatically less polarized among people who are particularly informed, trained, and motivated, such as physicians.

In the present work, we examine physicians’ beliefs about treatments for COVID-19, benchmark their polarization against that of lay adults, and provide evidence for two psychological mechanisms that give rise to polarized beliefs among both groups.

## Methods

We study two samples: a novel panel of 592 board-certified critical care physicians (“physicians”) and a sample of 900 adults recruited from an online panel (“laypeople”), all based in the United States. We focused on critical care physicians because they are important decision makers in the treatment of severe COVID-19 and because their day-to-day judgments are less influenced by patient preferences, compared to other physicians. Additional information about both samples and other methodological details are in *SI Appendix*.

We surveyed physicians in three phases between April 2020 and April 2022 and surveyed laypeople concurrently with the final physician survey. In each survey, physicians evaluated a clinical vignette about a severely ill COVID-19 patient and made decisions about which treatments to administer. For each treatment option, physicians reported beliefs about effectiveness and the quality of clinical evidence and made incentivized predictions about the proportion of their peers who made the same decision. Laypeople reported beliefs about treatments but did not make treatment decisions. All participants also reported beliefs about the effectiveness of COVID-19 vaccines and their support for vaccine mandates as well as a range of individual characteristics. Most estimates are based on data from Phase 3 surveys, administered in March and April 2022.

To investigate the role of information consumption on belief polarization, we asked participants in both samples to report how they consume news (e.g., print, social media, television) as well as their preferred cable news source (if applicable). Cable news consumption is a particularly plausible source of variation in public exposure to COVID-relevant information because partisans watch different cable news networks (4), and networks differed markedly in their coverage of hydroxychloroquine, ivermectin, and vaccination (9, 15, 16). Physicians also reported how they engage with scientific research.

To measure bias in the evaluation of information, we embedded an experiment in surveys administered to both samples. Participants read an abridged research abstract (physicians) or a research summary written in a journalistic style (laypeople), both of which reported the results of the TOGETHER trial (17), a well-powered randomized controlled trial that failed to find evidence that ivermectin was effective for treating COVID-19.^*^ Between subjects, we randomized whether the treatment was identified as ivermectin or was anonymized (“GL-22”). We then elicited beliefs about the study’s informativeness, its methodological rigor, and the likelihood that its authors were biased. We expected partisans’ beliefs on all three measures to diverge more sharply when the drug was identified as ivermectin.

**Fig. 1.**
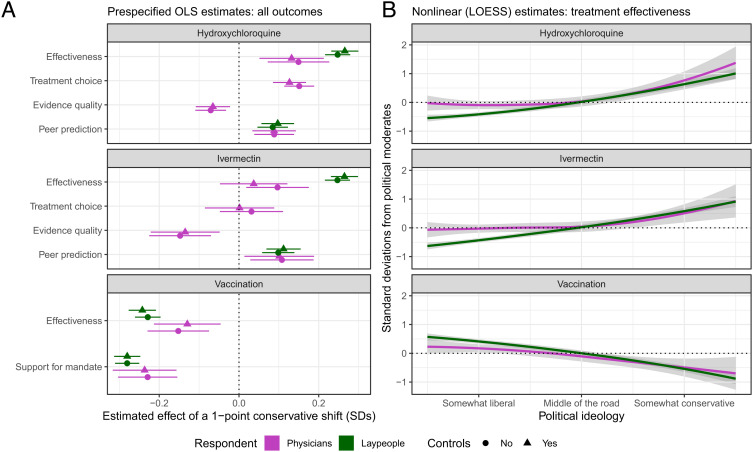
(*A*) Values are standardized beta coefficients from ordinary least squares (OLS) regressions, representing the estimated effect of a one scale-point conservative shift in political ideology, expressed in standard deviations from the mean response of political moderates. Controls are age, gender, education*, practice setting**, time spent in a clinical capacity**, base clinical specialty**, engagement with scientific research**, log population density (zip code level), census metropolitan classification (census tract level), and Republican vote share (2016, county level). Horizontal lines denote 95% confidence intervals. *Laypeople only. **Physicians only. (*B*) Predicted values from LOESS regressions. Shaded areas are 95% confidence intervals.

We measure political ideology on a 7-point scale bounded by “very liberal” and “very conservative,” with the midpoint defined as “middle of the road” (18). To compare polarization across samples and outcomes, we standardized all outcome variables by subtracting the mean response of political moderates (“slightly liberal,” “middle of the road,” “slightly conservative”) from each response and dividing by the standard deviation.^†^ Our primary analyses control for a range of plausible confounding factors, including demographic, professional, and regional characteristics (Fig. 1*A* caption).

For simplicity and statistical power, we collapsed measures that were collected at multiple time points after ruling out significant temporal variation (ANOVAs; ps > 0.18).^‡^ The timing of key elicitations can be found in *SI Appendix*, Table S1. Full study materials are at https://github.com/pitt-healthsciences/covid_polarization/. A preregistration specifying some elements of our analytical approach can be found at https://aspredicted.org/11M_35D. Following our prespecified exclusion criteria, we retained 410 physician responses and 882 layperson responses. Research was approved by the University of Pittsburgh Institutional Review Board. All subjects provided informed consent.

## Results

We find robust evidence of polarization on eight of ten physician outcomes and all six layperson outcomes (ps < .001; Fig. 1*A*). To illustrate the magnitude of these effects, conservative physicians were approximately five times more likely than their liberal and moderate colleagues to say that they would treat a hypothetical COVID-19 patient with hydroxychloroquine (Cohen’s h = .37). On average, physicians’ beliefs were less polarized than laypeoples’ (${\overset{¯}{\beta }}_{\mathrm{physician}}=.129;\;{\overset{¯}{\beta }}_{\mathrm{layperson}}=.204$). This difference was driven in large part by agreement between liberal and moderate physicians, with conservative physicians displaying polarization that was often comparable to that of conservative laypeople (e.g., Fig. 1*B*).

We next turn to evidence regarding psychological mechanisms underlying polarization, starting with the role of information exposure. Consistent with prior work (4, 19, 20), we find that liberals and conservatives consume different cable news, with conservatives being dramatically more likely to prefer Fox News. The preference for Fox News is a significant predictor of eight outcomes (controlling for political ideology). For these eight, adding cable news preferences to our preregistered models attenuates the estimated effect of political ideology on outcomes (by an average of 28%)—consistent with partial mediation for six outcomes and with either full or partial mediation for two.^§^ We note that estimates of the effect of cable news preferences on outcomes likely capture both causal effects of information exposure (9, 19) and endogenous characteristics, which are causes of both watching and outcomes. We do not find that political ideology is associated with the reported medium of news consumption or with how physicians engage with scientific evidence.

**Fig. 2.**
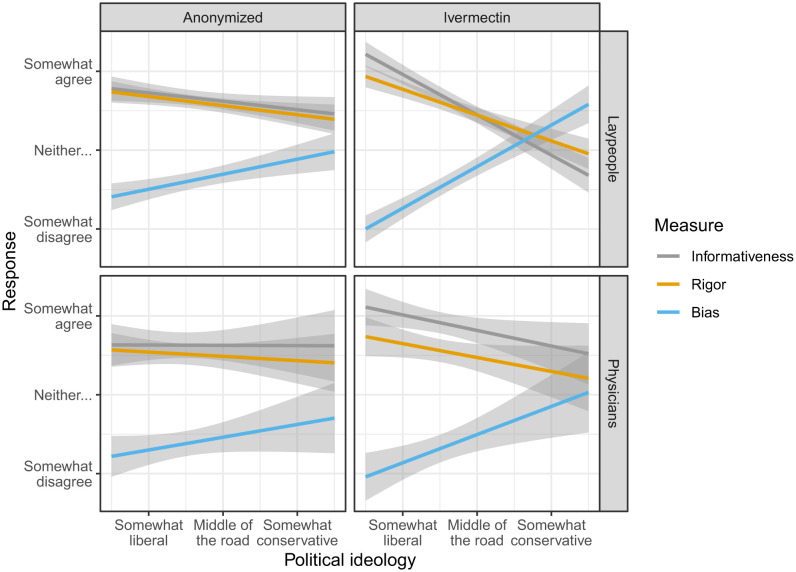
Experimental results. Values are predicted responses from OLS regressions. Shaded areas are 95% confidence intervals. All measures were elicited on 5-point scales bounded by “strongly agree” and “strongly disagree.”

The results of our experiment reveal that political ideology colors the evaluation of scientific evidence to a greater degree when it pertains to a politicized treatment. After reading otherwise identical results, partisans’ responses were more polarized when the drug was identified as ivermectin relative to when it was anonymized, with participants who were more conservative reporting that the evidence was less informative, the study was less methodologically rigorous, and the authors were more likely to be biased (ps < .001; Fig. 2).^¶^ These results were not detectably different across lay and physician samples (ps > .236), but our point estimates suggest that the experimental condition had a smaller effect on physicians’ responses, and our primary analyses of physicians’ responses alone were not conclusive.

## Discussion

Why are politically polarized beliefs so pervasive? In the context of COVID-19, our results suggest that it is not for lack of knowledge or motivation. Rather, we find strong evidence of belief polarization about COVID-19 treatments even among highly trained professionals making informed judgments within their area of expertise. Our findings are consistent with at least two explanations for this polarization: 1) that partisans consume different information and 2) that they evaluate the same information in a politically biased manner.

Our study makes several important advances. First, while prior work has examined polarization in physicians’ attitudes about value-laden topics such as abortion and gun ownership (21), we identify polarization of physicians’ beliefs about scientific facts and the clinical decisions that result from those beliefs. We also identify when and how polarization differs between physicians and laypeople.

These results also provide insight into the causes of regional variation in the use of hydroxychloroquine and ivermectin, both of which have been prescribed by physicians at much higher rates in conservative counties (22, 23). Our findings suggest that this variation is likely driven in part by the beliefs of physicians rather than by patient preferences alone.

Finally, our work highlights the limits of expertise and exposure to scientific evidence in mitigating polarization, underscoring the need for psychologically informed interventions to improve evidence dissemination on politicized topics. Such interventions might draw on bipartisan sources (24, 25), appeal to shared values (26, 27), or highlight implications that are desirable to liberals and conservatives alike (28).

## Supplementary Material

Appendix 01 (PDF)

## Data Availability

Data and materials are available at https://github.com/pitt-healthsciences/covid_polarization.

## Footnotes

^*^We focused on the TOGETHER trial because it had not yet been published in the peer-reviewed literature at the time of survey administration and therefore participants would be less likely to recognize the results, compared to earlier-published trials of other politicized treatments such as hydroxychloroquine.

^†^Note that this approach is identical to Z-scoring, with the only difference being that we subtract the mean of a predetermined group (moderates) instead of the overall mean. This approach improves coefficient interpretability without affecting the results of any statistical tests.

^‡^This step was not preregistered but does not substantively affect any estimates.

^§^*SI Appendix* sections “Mediation” and “Multicollinearity” contain additional information about these analyses.

^¶^These results are based on OLS models, regressing each outcome variable on the interaction between political ideology and a dummy variable for experimental condition.
