# Supplementary material for: The political polarization of COVID-19 treatments among physicians and laypeople in the United States
Source: Proc Natl Acad Sci U S A. 2023 Feb 8;120(7):e2216179120. doi: 10.1073/pnas.2216179120 (PMC9963903; doi:10.1073/pnas.2216179120)
Supplement: Supplementary file 1 — Appendix 01 (PDF) [file pnas.2216179120.sapp.pdf]

Supplementary materials: The political polarization of COVID-19 treatments among physicians and laypeople in the United States

Joel Levin, Leigh Bukowski, Julia Minson, and Jeremy Kahn

Contents

Materials **1**
Clinical vignette: Physicians . . . 1
Research abstract: Physicians . . . 2
Research summary: Laypeople . . . 2

Sample characteristics **2**
Physicians . . . 2
Laypeople . . . 2

Measures **3**
Survey . . . 3
Experiment . . . 4
Covariates . . . 4

Timing of elicitations **6**

Mediation **6**

Multicollinearity **7**

Other methodological notes **8**
Within-participant variation in reported political ideology . . . 8
Preregistration timing . . . 8

Materials[1]

Clinical vignette: Physicians

In each Phase, physicians evaluated a clinical vignette about a patient with COVID-19. For all participants, we randomized the patient's age (55, 65, 75, or 85) and gender (male or female). Randomization was intended to make our results more generalizable and we did not analyze the effects of these randomizations in any analyses. The text of the vignette is as below:

> A previously healthy [age] year old [man/woman] is admitted to the ICU from the emergency department after presenting with fever, dry cough, and shortness of breath. Yesterday, [he/she] was seen in an outpatient clinic where [he/she] tested positive for the SARS-CoV-2 infection (i.e. COVID-19) and was told to self-quarantine.
>
> In the emergency department, [he/she] experienced progressive dyspnea and was intubated for respiratory failure. The patient takes no medications at home, there are no contraindications to

[1] Full materials (and data) are available at https://github.com/pitt-healthsciences/covid_polarization

any specific medicines, and the EKG is normal. Enrolling [him/her] in a clinical trial is not an option.

Research abstract: Physicians

In Phase 3, physicians evaluated a research abstract as part of our experiment. The text of the abstract is as below:

> Please read and evaluate the following abbreviated abstract.
>
> **Methods**: As part of an ongoing platform trial, patients with a recent positive test for COVID-19 were randomized 1:1 to receive either [drug] (400mcg/kg daily for 3 days) or placebo. The primary endpoint was hospitalization within 28 days of randomization.
>
> **Results**: Assignment to the [drug] arm was stopped for futility after enrolling 1355 patients. At 28 days, 12.8% of patients in the [drug] group had been hospitalized (95% CI, 10.4% to 15.4%) compared to 14.1% in the placebo group (95% CI, 11.6% to 16.8%). The relative risk of hospitalization for patients in the [drug] group was 0.91 (95% CI, 0.69 to 1.19; posterior probability of superiority = .76).

Research summary: Laypeople

In their survey responses (concurrent with Phase 3 of the physician survey), laypeople evaluated a research summary as part of our experiment. The text of the summary is as below:

> Please read and evaluate the following research summary.
>
> A recent clinical trial randomized 1355 people who tested positive for COVID-19 to receive either [drug] or a placebo. After 28 days, 12.8% of the people who received [drug] had been hospitalized, compared to 14.1% of patients who received a placebo.
>
> The difference between these two percentages was not statistically significant, which means that the difference was likely due to random chance. On this basis, the researchers concluded that their data did not support [drug] as an effective treatment for COVID-19.

Sample characteristics

Physicians

All physician participants are board-certified United States intensivist physicians and were recruited by email from a list of physicians maintained by the American Medical Association. Initial recruitment took place in April of 2020. The sample is 24.9% female, has a mean age of 45 (SD = 9), and is 69% white. The modal participant has a base specialty of internal medicine (75%) and practices in an academic hospital (63%). The sample is politically diverse: 55% liberal, 25% “middle of the road”, and 20% conservative.

Laypeople

Lay participants were recruited using Prolific Academic and were matched to the US population on age, sex, and ethnicity using data from the US Census Bureau. Recruitment took place in March and April of 2022. We recruited in three waves of 300 participants each to match the timing of our recruitment emails to physicians. The sample is 49.3% female, has a mean age of 45 (SD = 16), and is 72% college educated. 55% of lay participants are liberal, 18% are “middle of the road,” and 26% are conservative.[2]

[2] These percentages do not sum to 100 because of rounding.

Measures

Survey

Treatment choice "Would you treat this patient with [drug]?"

- Definitely would not (-2)
- Probably would not (-1)
- Probably would (1)
- Definitely would (2)

Evidence quality "In your opinion, what is the quality of the empirical evidence about whether or not [drug] is effective for COVID-19?"

- Lowest quality (-2)
- Low quality (-1)
- Moderate quality (0)
- High quality (1)
- Highest quality (2)

Effectiveness "In your opinion, is [drug] an effective treatment for COVID-19?"

- Definitely not effective (-2)
- Probably not effective (-1)
- Probably effective (1)
- Definitely effective (2)

Effectiveness (vaccine) "COVID-19 vaccines are generally safe and effective for preventing severe illness."

- Strongly disagree (-2)
- Somewhat disagree (-1)
- Neither agree nor disagree (0)
- Somewhat agree (1)
- Strongly agree (2)

Support for vaccine mandate "Public authorities should seriously consider mandating vaccination against COVID-19 for adults who do not have a medical or religious exemption."

- Strongly disagree (-2)
- Somewhat disagree (-1)
- Neither agree nor disagree (0)
- Somewhat agree (1)
- Strongly agree (2)

Peer prediction (physicians) "You indicated that you (would / would not) treat the COVID patient with [drug]. What percentage of other physicians taking this survey do you think made the same choice?"

- Slider from 0-100 with endpoints labeled "None of them" and "All of them."

We linked participants to a page that further explained the definitions of terms in this question and the specifics of our incentive scheme. You can view that page by **clicking here**.

Peer prediction (laypeople) "You said that [drug] (was / was not) effective for treating COVID-19. What percentage of other participants in this survey do you think agreed with you?

(The other participants are US residents on Prolific Academic.)"

- Slider from 0-100 with endpoints labeled "None of them" and "All of them".

Note that the peer prediction questions are different for physicians and laypeople because we did not ask laypeople to make a clinical choice.

Both peer prediction questions were re-coded based on participants' responses to previous questions, such that they represent participants' predictions about the proportion of their peers who said that they would treat a patient with the drug in question (physicians) or that they believed that the drug in question was effective (laypeople).

Experiment

Items:

- **Informativeness** "This trial provides compelling evidence that [drug] is not effective for COVID-19."
- **Rigor (physicians)** "This trial is methodologically rigorous."
- **Rigor (laypeople)** "This trial used strong scientific methods."
- **Bias** "There is reason to suspect that the researchers were motivated to find a particular result."

Response options:

- Strongly disagree (-2)
- Somewhat disagree (-1)
- Neither agree nor disagree (0)
- Somewhat agree (1)
- Strongly agree (2)

Covariates

Percentages of respondents (final sample) are in parentheses.

We treat all variables with categorical responses as categorical variables (i.e., each category is dummy coded and entered into regression models as an individual variable), with the exception of *engagement with scientific research*, which we treat as a count variable. For *engagement with scientific research*, treating each response option as its own dummy variable does not affect our key estimates.

Education (laypeople) "What is the highest level of education you have completed?"

- Less than high school (0.4%)
- High school or equivalent (15.5%)
- Some college (0%)
- 2 year degree (12.1%)
- 4 year degree (48.9%)
- Graduate or professional degree (19.6%)
- Doctorate (3.5%)

Practice setting (physicians) "Which of these best describes your practice setting?"

- Academic, university affiliated (62.9%)
- Academic, not university affiliated (10.5%)
- Community (25.9%)
- Other (0.7%)

Time spent in a clinical capacity (physicians) "Over the past year, what proportion of your professional time was spent in direct practice of critical care?"

- All or almost all (95% or more) (32.9%)
- More than 50% but less than 95% (32.7%)
- Less than 50% (33.4%)
- None (1.0%)

Base clinical specialty (physicians) "What is your base specialty?"

- Internal medicine, not pulmonary (10.5%)
- Internal medicine, pulmonary (64.4%)

- Emergency medicine (3.7%)
- Anesthesiology (10.2%)
- Surgery (6.6%)
- Neurology (0%)
- Other (4.6%)

Engagement with scientific research (physicians) "In your effort to keep up with the medical literature, which of the following activities did you do in the last week? (Select all that apply.) We realize that you may do all of these at different times — we are interested in which of these you did only in the last week."

- Followed a discussion of a new paper on social media (e.g. Twitter)
- Read a summary of an original research article (e.g. Journal Watch)
- Read the table of contents of a medical journal
- Read the abstract of an original research article in a medical journal
- Looked through the full text of an original research article in a medical journal
- Closely read the full text of an original research article in a medical journal
- Discussed the results of an original research article with a friend or colleague

Timing of elicitations

"P" denotes physician responses, "L" denotes lay responses.

	Phase 1	Phase 2	Phase 3
**Hydroxychloroquine**			
Treatment choice	P	P	P
Evidence quality	P	P	P
Peer prediction	P	P	P/L
Effectiveness			P/L
**Ivermectin**			
Treatment choice			P
Evidence quality			P
Peer prediction			P/L
Effectiveness			P/L
**Vaccination**			
Effectiveness			P/L
Support for mandate			P/L
**Evidence evaluation experiment**			P/L
**Individual characteristics**			
Demographics	P		L
Professional characteristics	P	P	P
Political ideology		P	P/L

Mediation

We assess mediation using the Baron & Kenny method (this is a link) and adopt their naming conventions. All models are OLS regressions and include the full set of prespecified controls ("PSC").

- Path `C` is the *total effect* of political ideology ("`politics`") on outcomes: `outcome = politics + PSC`
- Path `A` is the effect of politics on the preference for Fox News ("`Fox`"): `Fox = politics + PSC`
- Path `B` is the effect of the preference for Fox News on outcomes, controlling for political ideology: `outcome = Fox + politics + PSC`
- Path `C'` ("C prime") is the *direct effect* of political ideology on outcomes, controlling for the preference for Fox News: `outcome = politics + Fox + PSC`

We define results as follows:[3]

- If path `C` is not signicant, there is no effect for which to evaluate the presence of mediation.
- If path `C` is significant but either or both of paths `A` and path `B` is not, there is no mediation.
- If paths `C`, `A`, and `B` are significant, the coefficients on paths `C` and `B` have the same sign, and we can reject the null that the coefficient on politics in path `C'` is zero, there is partial mediation.
- If paths `C`, `A`, and `B` are significant, the coefficients on paths `C` and `B` have the same sign, and we can**not** reject the null that the coefficient on politics in path `C'` is zero, there is either full mediation or partial mediation.[4]

The preference for Fox News either fully or partially mediated the magnitude of the relationship between politics and two outcomes. Those outcomes are:

[3]"Significant" refers to p values $< .05$.

[4]Note that this pattern is often referred to simply as "full mediation," even though models that produce this pattern are often not statistically distinguishable from models that produce the previous pattern, as is the case in our analyses.

- Physicians' beliefs about whether hydroxychloroquine is effective (57% attenuation)
- Physicians' beliefs about the proportion of their peers who said they would administer ivermectin (48% attenuation)

The preference for Fox News partially mediated the relationship between politics and six outcomes. Those outcomes are:

- Physicians' choices about whether to administer to administer hydroxychloroquine (39% attenuation)
- Physicians' support for a vaccine mandate (34% attenuation)
- Laypeoples' beliefs about the effectiveness of hydroxychloroquine (20% attenuation)
- Laypeoples' beliefs about the effectiveness of ivermectin (17% attenuation)
- Laypeoples' beliefs about the effectiveness of vaccination (10% attenuation)
- Laypeoples' support for a vaccine mandate (6% attenuation)

Multicollinearity

Political ideology (hereafter: "`politics`") and cable news preferences (hereafter: "`Fox`," a dummy variable that takes a value of 1 when the respondent indicated a preference for Fox News, 0 otherwise) are very strongly correlated (r = 0.52; Cohen's d = 2.35), consistent with prior work. This collinearity does not affect our primary analyses, which do not include `Fox` as a control, but has the potential to affect the interpretation of our mediation analyses, which are based in part on models which include both variables, as noted in the previous section.

The primary concern related to multicollinearity in this context is that it reduces the precision of estimates that come from models which include both `Fox` and `politics`. To assess the magnitude of imprecision resulting from multicollinearity, we compared standard errors for estimates of `Fox` and `politics` as a function of whether we included the other variable in each model. For the below reported results, all models included the full set of preregistered controls.

Across all 16 outcomes (10 physician outcomes, 6 layperson), introducing the collinear variable increased the average standard error of our estimates of `politics` from .030 to .035 (17%)[5] and increased the standard errors of our estimates of `Fox` from .171 to .191 (12%), reflecting a relatively modest impact of multicollinearity on precision.

The primary impact of this imprecision is to bias test statistics for mediation paths `B` and `C'` (and only these paths) towards the null, increasing the probability of type II error.

For path `B`, imprecision would lead us to erroneously conclude that Fox does not affect outcomes, ruling out mediation that actually exists. In the manuscript, we report that `Fox` (fully or partially) mediates the effect of `politics` on eight of our outcomes. Considering the potential impact of multicollinearity on precision, we should conclude that eight is the lower bound of the true number of (fully or partially) mediated outcomes.

The effect on path `C'` is only relevant if we conclude that there is mediation (i.e., that the key coefficients in paths `C`, `A`, and `B` are all significantly different from zero). In these cases, imprecision would lead us to erroneously conclude that mediation is full rather than partial. In the manuscript, we report that `Fox` fully mediates the effect of `politics` on two of those eight outcomes. Considering the potential impact of multicollinearity on precision, we should conclude that two is the upper bound of the true number of fully mediated outcomes.

In short, imprecision resulting from multicollinearity would tend to cause us to understate the number of outcomes for which the preference for Fox News mediates the effect of political ideology.

[5]This change also captures the impact of a reduction in sample size. This occurs because we only ask the question about cable news preferences during Phase 3, and therefore regressions that include `Fox` drop observations from participants who did not respond to the Phase 3 survey.

Other methodological notes

Within-participant variation in reported political ideology

Because we capture political ideology for some physicians twice (during Phases 2 and 3), there are cases in which we have two different measures of political ideology for the same participant. This is true of 39% of those participants who responded two both Phases 2 and 3. We could not identify any patterns in these changes: from Phase 2 to Phase 3, participants were equally likely to report becoming more conservative (20%) as they were to report becoming more liberal (19%) and only 3% changed their response by more than one scale point. We believe that this pattern is consistent with (random) measurement error and therefore chose to take the average of the two measurements whenever they were not the same. None of our reported results differ meaningfully when we use only our initial (Phase 2) or most recent (Phase 3) measurement of political ideology instead of the average of the two.

Preregistration timing

We note that our preregistration was submitted after data for Phases 1 and 2 had already been collected, and therefore that it applies only to Phase 3 analyses and results.
